# Involvement of PPARγ/FSP27 in the pathogenic mechanism underlying insulin resistance: tipping the balance between lipogenesis and fat storage in adult catch-up growth rats

**DOI:** 10.1186/s12986-019-0336-9

**Published:** 2019-02-11

**Authors:** Yan Li, Shan Yu, Lulu Chen, Xiang Hu, Juan Zheng, Xiuling Deng

**Affiliations:** 1 Department of Endocrinology, Heping Hospital Affiliated to Changzhi Medical College, Changzhi, 046000 People’s Republic of China; 2 Department of anesthesiology, Union Hospital, Tongji Medical College, Huazhong University of Science and Technology, Wuhan, 430022 People’s Republic of China; 3 Department of Endocrinology, Union Hospital, Tongji Medical College, Huazhong University of Science and Technology, Wuhan, 430022 People’s Republic of China

**Keywords:** Catch-up growth in adult, Visceral fat accumulation, Fat storage capacity, Lipid overflow, Insulin resistance

## Abstract

**Background:**

Catch-up growth in adult (CUGA) is characterized by visceral fat accumulation, ectopic lipid deposition and insulin resistance (IR). Here, we investigated the determinants of these pathophysiological consequences of CUGA.

**Methods:**

Rats were divided into different groups: control rats were offered normal chow ad libitum (AL), while experimental rats were put on 4-week caloric restriction (CR) initially, followed by regaining weight-matched normal chow (RN) in the RN group. General characteristics of lipid metabolism, expression level of genes in visceral adipose tissue (VAT), and glucose infusion rate (GIR_60–120_) by the hyperinsulinemic-euglycemic clamp were examined.

**Results:**

After CR, percentage of abdominal fat mass (AFM%) was lower in the RN group than in the AL group but no difference was observed in serum non-esterified fatty acid (NEFA). Expression of fat-specific protein 27 (FSP27) was decreased in the RN group, while the expression of peroxisome proliferator-activated receptors γ (PPARγ), the key lipogenic gene, was increased. After refeeding, AFM% increased over time and serum NEFA persistently elevated in the RN group. Ectopic triglyceride contents were increased whereas insulin sensitivity was impaired. The expression of FSP27 did not follow the increase in the expression of PPARγ. Additionally, we observed a sustained increase in the expression of ATGL and CGI-58 in VAT in the RN group compared with the AL group after CR and refeeding, and a persistent shift-to-the-left of adipocyte size distribution accompanied by enhanced lipogenesis during CUGA.

**Conclusion:**

The persistent CR-induced imbalance of lipogenesis/fat storage capacity might be responsible for the CUGA-associated metabolic disorders.

## Introduction

Nowadays, insulin resistance (IR)-associated diseases present an explosive increase in prevalence in developing countries, especially in Asia [1]. It is reported that developing countries have widely undergone rapid transition in nutritional status, which usually leads to catch-up growth (CUG) [2, 3]. Recent researches have emphasized that CUG, characterized by catch-up accumulation of fat, is a principal risk factor for IR-associated diseases later in life [4]. Therefore, CUG is extremely important to account for the epidemiological changes of IR-associated diseases in developing countries [1, 5].

Employing a rat model of catch-up growth in adult (CUGA) developed by caloric restriction (CR)-weight matched refeeding, we have previously observed that CUGA leads to visceral fat accumulation, ectopic lipid deposition and drastic IR within a short time during refeeding after CR [5–7]. This raises a question as to why CUGA rats without high-fat diet exhibit phenotypes of classical IR. Although the precise mechanisms involved still remain poorly understood, recently, emerging evidences have revealed that CR remarkably enhances lipogenic potential but attenuates fat storage capacity [8, 9]. Fat storage capacity is the capacity of the adipocytes to accumulate triglyceride stores within the cells. It can be directly measured or indirectly assessed based on adipocyte morphology or X-ray imaging [8, 9].

Ectopic lipid deposition has been recognized as a critical pathogenic factor of IR [10–12]. Once increase in fat storage capacity is not in parallel with the increase in lipogenesis, it will most likely result in lipid spillover, ectopic lipid deposition and IR [13, 14]. One of the mechanisms may be white adipose tissue (WAT) dysfunction with lipid deposition [13, 15]. This is supported by findings showing that fat storage capacity of WAT in obese insulin-sensitive human subjects was elevated compared with obese insulin-resistant human subjects [14, 16, 17].

In mammals, WAT is currently considered as the main lipid storage depot, and is of crucial importance in modulating whole-body lipid flux [13, 15]. WAT exerts its regulatory action by efficient conversion of surplus nutrients to triglyceride, then storing triglycerides in lipid droplets (LD), and releasing fatty acids into the circulation through breaking down the stored triglycerides [15]. This metabolic flexibility in WAT is critical for whole-body lipid homeostasis [16, 18–20]. The lipogenic capacity of WAT is controlled mainly by PPARγ [21], while the storage capacity is regulated principally by FSP27 [22]. PPARγ belongs to the nuclear hormone receptor superfamily and can promote de novo lipogenesis by regulating target genes like fatty acid synthase (FAS) and lipoprotein lipase (LPL) [21]. It plays an important role in the lipogenesis of adipocytes [23]. FSP27 is localized on the surface of LD and is one of the LD-associated proteins [22]. It is a crucial factor for the regulation of the storage function of adipocytes [24]. Recent studies also reported that perilipin1 is required for FSP27-mediated promotion of lipid storage in unilocular LD of adipocyte through increasing lipid exchange and transfer. Decrease in perilipin1 expression partly impairs fat storage mediated by FSP27 [25]. This capacity of fat storage also can be reflected by adipocyte size [26]. As FSP27 expression is under the control of PPARγ [14], the efficient fat storage in WAT depends on the concerted expression of FSP27 and PPARγ [16]. Therefore, we evaluated the relative changes in lipogenesis and fat storage capacity through the change in expression of PPARγ and FSP27. Additionally, a recent study proposed that the enzymatic capacity for fatty acid synthesis determined insulin-stimulated 2-DG uptake in WAT [27]. We postulated that FAS activity might be an important factor in determining insulin-mediated glucose uptake in VAT during CUGA [28].

Moreover, to determine whether the imbalance of lipogenesis/fat storage capacity has any effect on lipid overflow and ectopic lipid deposition, lipolytic capacity in visceral adipose tissue (VAT), serum non-esterified fatty acid (NEFA), and ectopic triglyceride contents are widely accepted as critical risk factors. Adipose triglyceride lipase (ATGL), also known as triglyceride hydrolase, is of importance in the dysregulation of lipolysis and lipid spillover in obesity as well [29, 30]. Although ATGL and hormone-sensitive lipase (HSL) are not tightly coordinated in response to drastic changes in food supply during CR, it is reasonable to believe that ATGL is the major lipase at least in CR [31]. Its activity is associated with LD-associated proteins and co-activator comparative gene identification 58 (CGI-58) [32]. Under physiological conditions, FSP27 acts to constitutively limit the LD presence of ATGL [33], while perilipin1 inhibits ATGL activity via binding CGI-58 [34]. Normal lipid storage function, thus, is an essential prerequisite for sequestering triglycerides in LD away from ATGL and preventing lipid spillover and ectopic lipid deposition [14]. Emerging evidence has demonstrated that deficiency in expression of FSP27 and perilipin1 along with up-regulation of ATGL and CGI-58 are involved in lipid overflow and the development of IR in obesity [14, 29]. Therefore, we also evaluated the relative changes in lipogenesis and fat storage capacity through the change in the expression of ATGL and CGI-58.

In brief, given the pathogenic significance of early CR for CUGA during refeeding [7, 35], the possibility arises that a sustained imbalance of lipogenesis/fat storage capacity during visceral fat accumulation in CUGA may be involved in the pathogenesis of CUGA-associated ectopic lipid deposition and IR. To test this hypothesis, this study investigated the changes in lipogenic and fat storage capacity in VAT, as well as lipid overflow, ectopic lipid deposition and IR during CUGA.

## Methods and materials

### Animals

Six-week-old male Sprague-Dawley (SD) rats (body weight 170 ± 10 g, *n* = 80) were obtained from SJA Laboratory Animal Co. Ltd. (Hunan, China) and maintained under SPF laboratory conditions in the Laboratory Animal Center of Tongji Medical College, Huazhong University of Science and Technology [36]. All protocols were approved by the Animal Ethics Committee of our university and in accordance with Hubei Province Laboratory Animal Care Guidelines for the use of animals in research.

### Experimental design

**Fig. 1:**
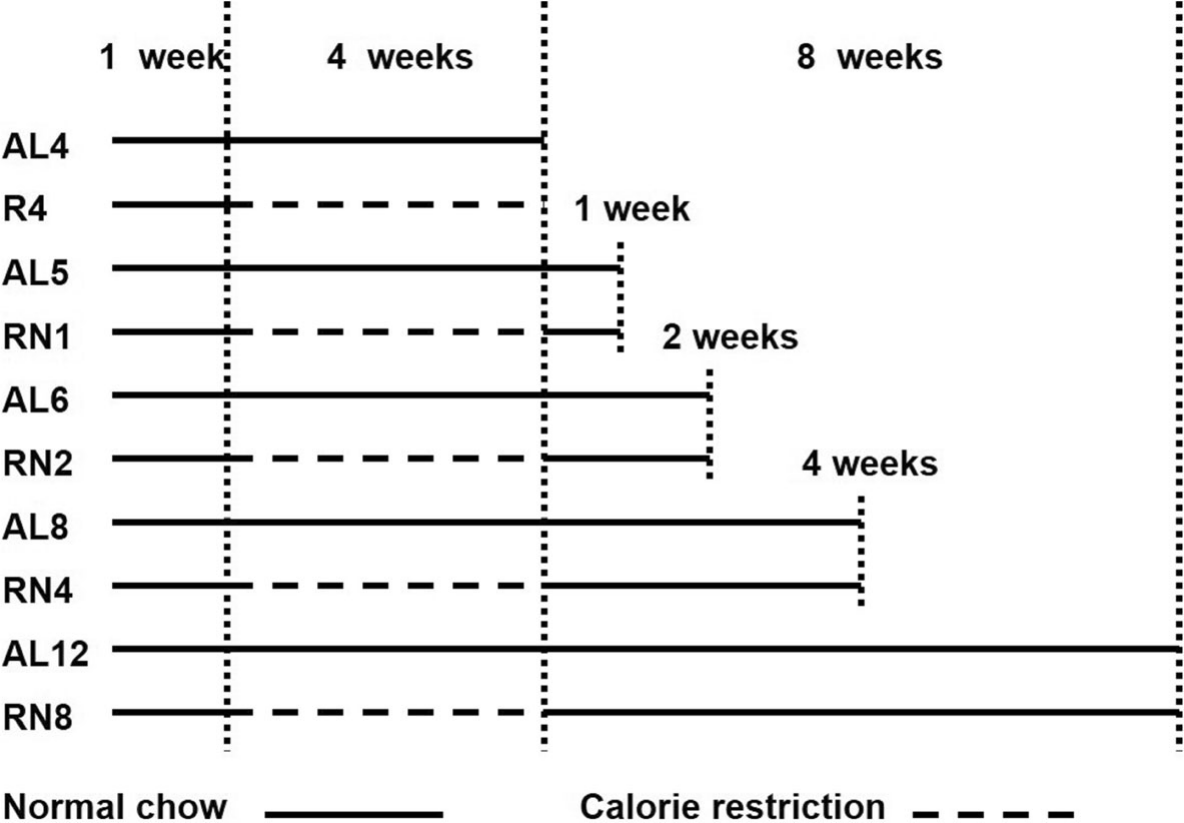
Experimental design. As mentioned in the method section, briefly, the rats were randomized into the AL and RN groups. Then, each group was divided into five subgroups for five different sampling points, with 8 rats in each subgroup. The AL groups (fed ad libitum with normal chow for 4, 5, 6, 8, or 12 weeks, i.e. AL_4_, AL_5_, AL_6_, AL_8_, and AL_12_, respectively) were age-matched controls of the RN groups. The RN groups were submitted to different feeding conditions and composed of a CR subgroup (fed with 60% of weight-matched normal chow for 4 weeks, also known as R_4_), and four CUGA subgroups (refed with weight-matched normal chow for 1, 2, 4, or 8 weeks after 4-week CR, i.e. RN_1_, RN_2_, RN_4_, and RN_8_, respectively)

The experimental design (Fig. 1) was employed as described previously [5, 6]. Briefly, following acclimation for 1 week, rats were randomized to the normal chow ad libitum (AL) and CUGA by normal chow (RN) groups based on body weight. Then, each group was divided into five subgroups for five different sampling points, with 8 rats in each subgroup. The AL groups (fed ad libitum with normal chow for 4, 5, 6, 8, and 12 weeks respectively, AL_4_, AL_5_, AL_6_, AL_8_, and AL_12_) were age-matched controls of the RN groups. The RN groups were submitted to different feeding conditions and composed of a CR subgroup (fed with 60% of weight-matched normal chow for 4 weeks, R_4_), and four CUGA subgroups (refed with weight-matched normal chow for 1, 2, 4, and 8 weeks respectively after 4-week CR, i.e. RN_1_, RN_2_, RN_4_, and RN_8_). The average daily food intake in each group was measured by the difference in weight between the amount of food provided and the amount of food remaining over a 1-day period, which was used to calculate energy intake [7].

### Serum assays

Serum NEFA was determined by a NEFA C kit (Wako Chemicals, Richmond, VA, USA). Fasting serum glucose was detected using a glucose oxidase kit (Roche Diagnostics GmbH, Rotkreuz, Switzerland). Serum insulin was quantified using a rat insulin ELISA kit (Millipore, USA).

### Body composition

Dual-energy X-ray absorptiometry (DEXA) scan was used to measured body composition, as published [37]. DEXA scan was performed using a GE Lunar Prodigy Advanced System (GE Healthcare, Milwaukee, WI, USA) to detect lean body mass (LBM), body fat mass (BFM) and abdominal fat mass (AFM). The data were analyzed by Encore 2007 Small Animal Software (version 11.40.004). AFM can be distinguished using DXA by identifying it as a specific region of interest within the analysis program. Fat mass from this region was strongly correlated with weighted visceral fat mass and had been validated to be a useful predictor of visceral fat mass.

### Hyperinsulinemic-euglycemic clamp and measurement of 2-deoxy-D-glucose uptake in vivo

A 120-min hyperinsulinemic-euglycemic clamp in conscious rats was carried out according to a protocol previously developed by our laboratory to evaluate systemic IR [5, 6]. The average glucose infusion rate was calculated in 60–120 min to reflect the degree of systemic insulin sensitivity. 45 min before the end of the clamp, 2-deoxy-D-glucose (Sigma-Aldrich, St Louis, MO, USA) was administered as a bolus (2 mmol/kg in saline) to evaluate insulin-stimulated glucose transport activity in VAT. The rate of 2-DG uptake into perirenal adipose tissue was measured using a non-radioisotope enzymatic assay [38].

### Sample collection

The rats were fasted for 10 h overnight and anesthetized by intraperitoneal injection of 10% chloral hydrate at 0.3 g/kg. The rats were quickly sacrificed after blood collection via cardiac puncture and the perirenal fat was quickly sampled.

### Triacylglycerol contents in peripheral tissues analysis

Triacylglycerol contents in liver and muscle were determined as previously published [39]. Briefly, 100 mg of frozen tissues (liver or muscle) were homogenized with 1 mL of chloroform/methanol (2:1, vol/vol). Next, the tissue homogenates were rotated at room temperature for 4 h to facilitate total lipid extraction. Then, 0.6% NaCl was added before centrifugation for 10 min. The bottom layer was collected, air dried, and resuspended in ethanol. The triacylglycerol concentration was quantified with an enzymatic colorimetric test kit (Triacylglycerol GPO-PAP, Roche Diagnostics, Indianapolis, IN).

### Fatty acid synthase activity measurement

FAS activities were measured in tissue lysates following previous instructions [40]. FAS activity was measured by mixing serial dilutions of FAS enzymes with the activity detection buffer containing 31.25 μM NADPH (Sigma, USA), 5 μM acetyl-CoA (Sigma, USA), 100 mM potassium phosphate (pH 7.0) and 1 mM DTT (Sigma, USA). The FAS assay was initiated by adding 10 μM malonyl-CoA (Sigma, USA). The conversion from NADPH to NADP was detected by the decrease in 340 nm absorbance using the SpectraMax M2 plate reader (Molecular devices, Sunnyvale, CA). Rates were normalized to the background rate of NADPH oxidation in the presence of acetyl-CoA. The specific FAS activity was expressed as nmol NADPH oxidized·min^− 1^·mg protein^− 1^.

### Measurement of adipocyte size

Sections from perirenal fat pat (*n* = 4 rats/group) were fixed in 10% formalin and paraffin-embedded. Hematoxylin/eosin staining was performed on 4-μm sections. Stained sections were scanned by inverted microscope attached to a cooled color CDD camera (Olympus IX71, Olympus Optical Co. Ltd., Tokyo, Japan). Eight digital images (100 ×) without overlapping field were taken from each slide (total of 32 fields per group) and adipocyte area was calculated using Image J software (NIH, Bethesda, USA).

### Quantitative PCR analysis

Total RNA was extracted from perirenal adipose tissues by RNeasy Mini Kit (Qiagen, Hilden, Germany), following the manufacturer’s instructions. cDNA was synthesized by using PrimeScript-RT reagent Kit (TAKARA, Shiga, Japan). Real-time quantitative PCR reactions were performed using SYBR-Premix Ex Taq (Takara) on an ABI 7500 real-time PCR System (Applied Biosystems, Foster City, CA, USA). Primers for different genes are described in Table 1.

**Table 1:** Nucleotide sequences of primers designed for quantitative PCR analysis

Gene	Sense primer (5′-3′)	Antisense primer sequence (5′-3′)
PPARγ	GCCCTTTGGTGACTTTATGGAG	GCAGCAGGTTGTCTTGGATGT
FAS	ACCTCATCACTAGAAGCCACCAG	GTGGTACTTGGCCTTGGGTTTA
FSP27	AAGGCATCATGGCCCACAG	TCTCCACGATTGTGCCATCTTC
Perilipin1	GTACACTATGTCCCGCTTCC	CCACCTCTGCTGGAGGATTA
ATGL	TGCGCAATCTCTACCGCCTCT	CGAAGTCCATCTCGGTAGCCCT
CGI-58	TGATCAAGAGAGACCAATTC	TCAGGCGCTGCACTAGACTC
HSL	ATCTGTGAAGAGGAAGCCTGAA	TAGTCTGTGAGGGTCTGCTGTG
LPL	CACAGTGGCTGAAAGTGAGAAC	GAGTCGTTCTTCCACTTAAGCTTC
β-actin	CACCCGCGAGTACAACCTTC	CCCATACCCACCATCACACC

### Immunoblotting

Perirenal adipose tissues lysates were prepared in ice-cold lysis buffer, as described previously [41]. Proteins were separated by 6–10% SDS-PAGE and blotted onto polyvinylidene difluoride membranes, blocked and then detected with the following primary antibodies: fat-specific protein 27 (FSP27), perilipin 1 (Abcam, Cambridge, UK), FAS, HSL, HSL phosphor-Ser^660^ (Cell Signaling Technology, Boston, MA, USA), peroxisome proliferator activated receptor- γ (PPARγ) and β-actin (Santa Cruz Biotechnology, Santa Cruz, CA, USA). The bands were quantified by scanning densitometry.

### Statistical analysis

Data were expressed as means ± SEM. Differences between the groups at the same sampling points were evaluated by unpaired student’s *t*-test. Distributions of adipocyte size were analyzed by the quantitative distribution method. The comparison of the distributions was determined by the Kolmogorov-Smirnov test. *P* < 0.05 was considered significant. Data were analyzed by SPSS software 13.0 (SPSS Inc., Chicago, IL, USA).

## Results

### Energy intake, body weight and visceral fat accumulation

**Fig. 2:**
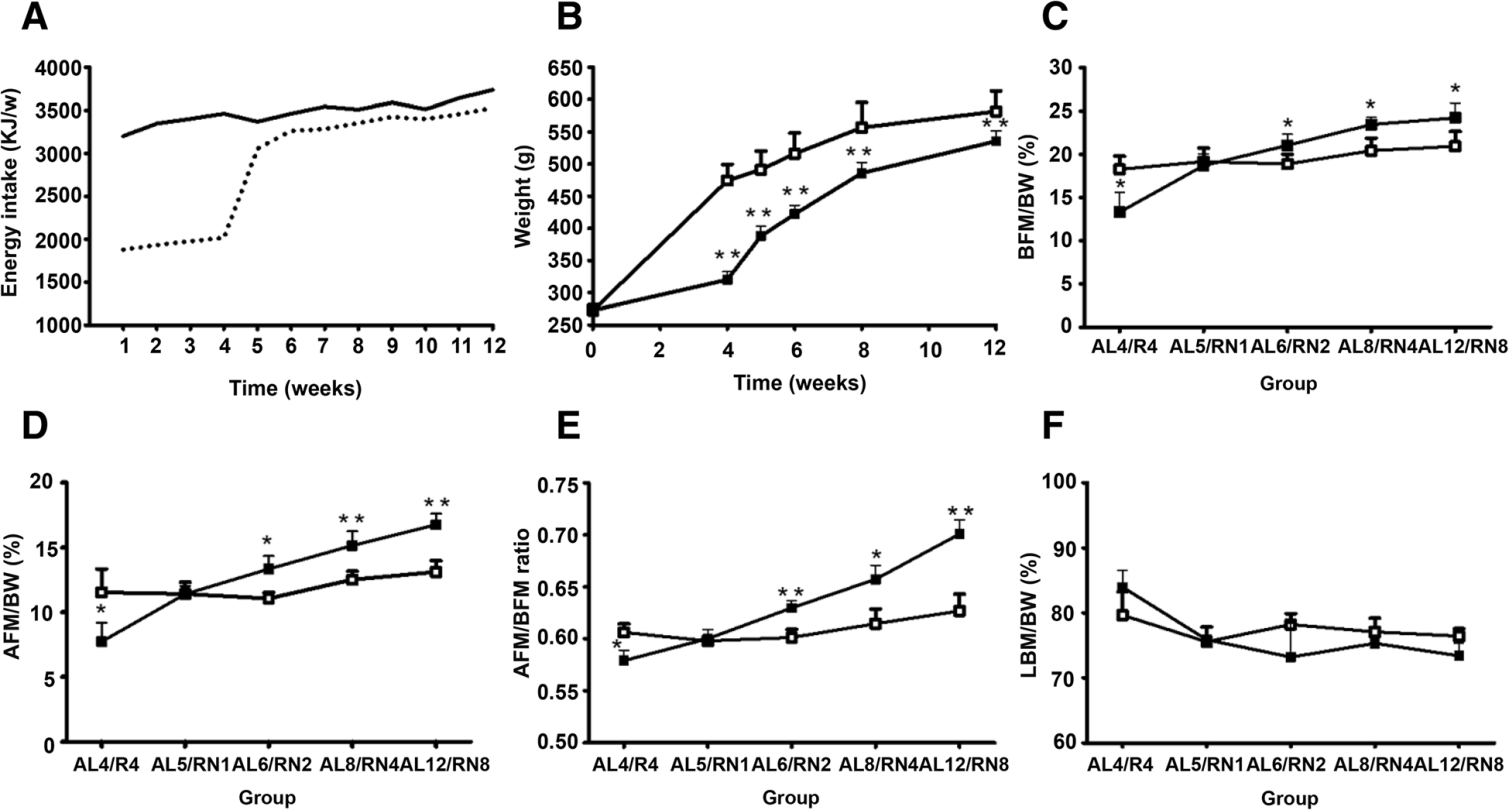
The rat model of catch-up growth in adulthood (CUGA) was modeled using a diet that matched the weight of the rats. The rats in the calorie restriction period were given a feed amount that was 60% of the free-fed volume of rats in the AL group with the same weight, while those in the diet recovery period were given a feed amount that was 100% of the free-fed volume of rats in the AL group with the same weight. Changes of (**a**) energy intake, (**b**) body weight, (**c**) BFM% (BFM/BW), (**d**) AFM% (AFM/BW), (**e**) AFM/BFM ration, and (**f**) LBM% (LBM/BW) over the course of CUGA (*n* = 8/group). Solid line/white square, AL groups; dotted line/black square, RN groups. *P < 0.05, **P < 0.01 versus the corresponding AL groups

As shown in Fig. 2a, during the experiment, the weekly energy intake of the rats in the RN group was lower than that of the AL group. During the caloric restriction period, the weekly energy intake of rats in the RN group was 60% that of rats with the same weight in the AL group. After restoring the diet, the weekly energy intake of the rats in the RN group quickly recovered to that of rats with the same weight in the AL group, but was slightly lower than that of the AL group (age matching). This resulted in a significant decrease in body weight (BW) (474.31 ± 25.11 for AL vs. 320.06 ± 12.40 for RN *P* < 0.05), BFM% (BFM/BW) (18.25 ± 1.54 for AL vs. 13.28 ± 2.28 for RN *P* < 0.05), AFM% (AFM/BW) (11.54 ± 1.81 for AL vs. 7.72 ± 1.47 for RN *P* < 0.05) and AFM/BFM ratio (0.61 ± 0.01 for AL vs. 0.58 ± 0.01 for RN *P* < 0.05) (− 32.5%, − 27.2%, − 33.1% and − 4.9%, respectively, *P* < 0.05) in the RN groups compared with the AL groups at the end of CR. After refeeding, energy intake in the RN groups was rapidly restored to the normal level of the weight-matched rats (Fig. 2a). However, BW in the RN groups was still remarkably lower than in the AL groups until 8 weeks of refeeding (581.78 ± 31.52 for AL vs. 535.60 ± 15.76 for RN *P* < 0.01; − 7.9%, *P* < 0.01) (Fig. 2b). In contrast, BFM% (19.14 ± 1.56 for AL vs. 18.67 ± 1.34 for RN *P* = 0.7), AFM% (11.41 ± 0.91 for AL vs. 11.40 ± 0.64 for RN *P* = 0.05) and AFM/BFM ratio (0.59 ± 0.01 for AL vs. 0.60 ± 0.01 for RN *P* = 0.2) in the RN groups succeeded in reaching the levels of the controls on week 1 of refeeding, and were significantly higher than controls on week 8 of refeeding (BFM%, 20.91 ± 1.72 for AL vs. 24.20 ± 1.71 for RN, 15.7%; AFM%, 13.11 ± 0.88 for AL vs. 16.77 ± 0.84 for RN, 27.9%; AFM/BFM, 0.63 ± 0.02 for AL vs. 0.70 ± 0.01 for RN, 11.8%; *P* < 0.05) (Fig. 2c-e). No significant differences were found in LBM% (LBM/BW) between the RN and AL groups before (79.65 ± 3.60 for AL vs. 83.97 ± 2.60 for RN *P* = 0.163) and after (76.41 ± 1.15 for AL vs. 73.39 ± 1.93 for RN *P* = 0.095 after 8 weeks refeeding) refeeding (Fig. 2f).

### Blood parameters, insulin sensitivity and ectopic lipid accumulation

As shown in Table 2, at the end of CR, serum NEFA in the R_4_ group was similar to that of the AL_4_ group. After refeeding, serum NEFA was markedly increased in the RN groups compared with the AL groups (*P* < 0.05). No differences in serum glucose were detected between the RN and AL groups. Notably, although no significant differences were observed in serum insulin between the R_4_ and AL_4_ groups at the end of CR, there was a tendency for serum insulin to be higher in the RN groups after refeeding. On week 4 and 8 of refeeding, serum insulin in the RN_4_ and RN_8_ groups was significantly increased in comparison with controls (*P* < 0.01). Consistently, a striking reduction of GIR_60–120_ at hyperinsulinemic-euglycemic clamp was observed in the RN_4_ and RN_8_ groups relative to their controls (*P* < 0.05). The HOMA-IR index was also increased in the RN groups, supporting the clamp results. Meanwhile, we also found that both intramuscular and intrahepatic TG contents were significantly increased in the RN_4_ and RN_8_ groups compared to the AL controls (*P* < 0.01). In brief, with the increase in NEFA during refeeding, RN rats showed a gradual increase in ectopic triglycerides and serum insulin level, but decrease in whole-body insulin sensitivity. Thus, we postulated that because of adequate energy supply at the refeeding stage, the enhanced NEFA exceeded oxidation in peripheral tissues, which then caused triglyceride accumulation in peripheral tissues and the development of IR.

**Table 2:** Metabolic parameters, GIR_60–120_ at euglycemic clamp and ectopic triglyceride contents at the end of CR and during refeeding

	Calorie Restriction		Refeeding							
	AL_4_	R_4_	AL_5_	RN_1_	AL_6_	RN_2_	AL_8_	RN_4_	AL_12_	RN_8_
Serum NEFA (μmol/l)	357.8 ± 34.4	349.1 ± 21.6	344.8 ± 43.7	422.4 ± 19.8*	362.1 ± 17.2	564.6 ± 43.7^†^	360.6 ± 12.4	688.1 ± 33.7^†^	363.5 ± 20.4	758.6 ± 45.0^†^
Serum glucose (mmol/l)	4.90 ± 0.45	4.77 ± 0.35	4.93 ± 0.39	5.13 ± 0.24	5.03 ± 0.15	5.18 ± 0.28	4.95 ± 0.52	5.15 ± 0.66	4.87 ± 0.39	5.02 ± 0.30
Serum insulin (pmol/l)	104.6 ± 2.7	97.1 ± 10.0	94.2 ± 3.0	91.6 ± 9.6	97.4 ± 11.8	102.4 ± 3.1	100.8 ± 3.6	140.1 ± 11.5^†^	105.2 ± 5.3	163.6 ± 16.3^†^
GIR_60–120_(mg·min^−1^·kg^−1^)	23.47 ± 0.89	25.34 ± 1.16	22.15 ± 1.44	24.15 ± 1.12	21.77 ± 1.38	23.30 ± 0.94	21.45 ± 1.20	14.27 ± 1.06*	21.43 ± 1.05	12.00 ± 1.64*
HOMA-IR	3.17 ± 0.21	2.86 ± 0.15	2.93 ± 0.14	2.88 ± 0.21	3.06 ± 0.27	3.13 ± 0.12	3.16 ± 0.16	4.73 ± 0.14^†^	3.11 ± 0.08	5.15 ± 0.39^†^
Intramuscular triglyceride (mg/g tissue)	7.05 ± 1.34	6.24 ± 1.79	6.54 ± 1.47	6.68 ± 0.91	6.42 ± 1.15	7.04 ± 0.91	7.23 ± 0.89	10.13 ± 0.90^†^	7.44 ± 0.64	12.16 ± 1.39^†^
Intrahepatic triglyceride (mg/g tissue)	11.10 ± 1.94	9.66 ± 1.77	10.85 ± 1.90	10.97 ± 2.92	11.37 ± 1.85	15.96 ± 2.36	13.53 ± 2.55	30.13 ± 5.80^†^	11.47 ± 1.70	36.03 ± 3.68^†^

The data were represented as Mean ± S.E.M. n = 4–8/group. NEFA, nonesterified fatty acid; GIR_60–120,_ average glucose infusion rate_60–120_ in hyperinsulinemic-euglycemic clamp; *HOMA-IR*, homeostasis model assessment for insulin resistance; AL, ad libitum normal chow; RN, catch-up growth in adult with normal chow. Statistical differences between AL and RN groups at the same experimental time point are indicated as follows: *P < 0.05, ^†^*P* < 0.01

### Changes of key regulatory factors involved in lipogenesis and fat storage capacity in VAT during CUGA

**Fig. 3:**
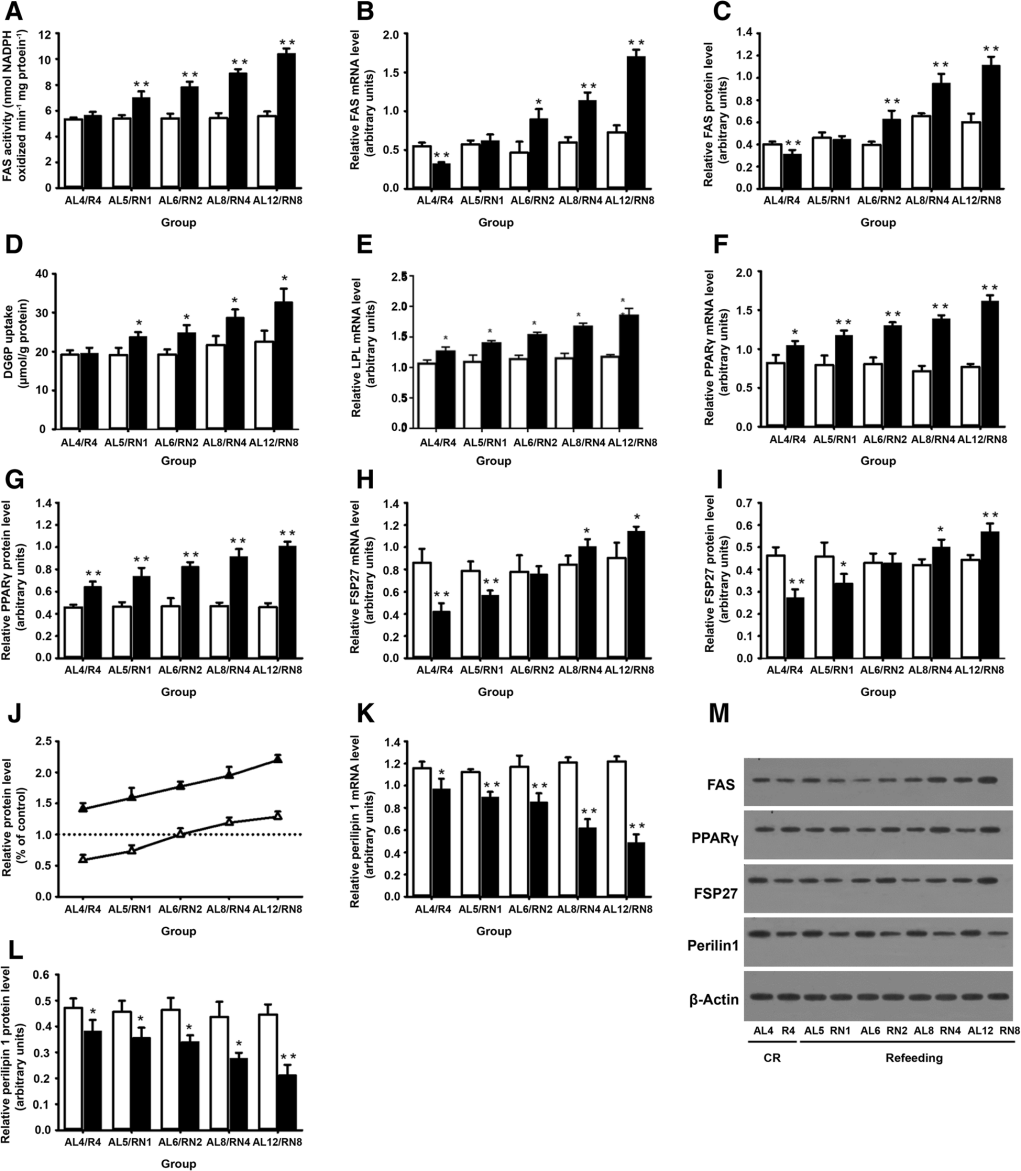
Lipogenesis and fat storage capacity in VAT during CUGA. **a** FAS activity and expression of FAS mRNA (**b**) and protein (**c**). **d** 2-DG uptake in respond to administration of insulin in hyperinsulinemic–euglycemic clamp. **e** Relative amounts of LPL mRNA . Analysis of PPARγ (**f**) and FSP27 (**h**) gene expression. Analysis of PPARγ (**g**) and FSP27 (**i**) protein expression. **j**The relative protein levels of PPARγ (black triangle) and FSP27 (white triangle) standardized by controls. **k** Gene expression of perilipin1. **l** Protein expression of perilipin1. **m** Representative immunoblot analysis for FAS, PPARγ, FSP27 and Perilipin1. β-actin served as a loading control. All experiments consisted of *n* = 4/group. White bars, AL groups; black bars, RN groups. *P < 0.05, **P < 0.01 versus the corresponding AL groups

FAS activity in the RN groups overshot controls after refeeding (*P* < 0.01), but there was no significant decrease at the end of CR (*P* > 0.05) (Fig. 3a). On the contrary, FAS gene expression was found to be markedly lower in the R_4_ group after 4 weeks of CR (*P* < 0.01), but after refeeding, FAS gene expression in the RN groups gradually increased over time. On week 2 of refeeding, FAS gene expression in the RN_2_ group was significantly up-regulated as compared with controls (*P* < 0.05) (Fig. 3b). A similar expression profile of FAS protein was observed (Fig. 3c and m). The results showed that although the increase in FAS activity is consistent with the elevation in FAS expression after 2 weeks of refeeding, the changes in FAS activity did not follow the changes in FAS expression at the end of CR and on week 1 of refeeding. Hence, the changes in FAS activity could only partially be explained by the changes in FAS expression, suggesting that additional posttranslational mechanisms are likely involved in the regulation of FAS activity. Interestingly, our results also showed that the change in insulin-mediated 2-DG uptake is paralleled by the change in FAS activity (Fig. 3d). Besides these, LPL has been considered a key regulators of lipid accumulation in adipocytes. As show in Fig. 3e, LPL gene expression significantly increased in response to 4 weeks of CR (*P* < 0.05), and it was more obvious in the RN groups than in their controls after refeeding (*P* < 0.05). Together, these results were in favor of the notion that during CUGA, enhanced lipogenesis might occur at the CR stage, and persisted after refeeding, which might be a pivotal factor for initiating and sustaining visceral fat accumulation.

Although the lipogenic capacity was enhanced, the triglyceride storage capacity in VAT was decreased relatively. The measurement of PPARγ gene expression indicated that it was increased by 0.41-fold in the R_4_ group compared with the AL_4_ group after CR (*P* < 0.05), and this was further aggravated by refeeding. At the end of experiment, PPARγ gene expression in the RN_8_ group was raised to 2.20-fold compared with the AL_12_ group (*P* < 0.01) (Fig. 3f). The PPARγ protein level was shown the similar tendencies (Fig. 3g, m). In contrast, FSP27 gene expression in the R_4_ group was dramatically reduced to 0.59-fold of the AL_4_ group by the end of CR (*P* < 0.01). However, refeeding only partially moderated this association. The lowered FSP27 gene expression in the RN groups was sustained till 2 weeks of refeeding. On week 8 of refeeding, FSP27 protein level in the RN_8_ group increased to 1.29 times that of controls (*P* < 0.01) (Fig. 3h, i, and m). The above results suggested that during CUGA, FSP27 gene expression lagged far behind PPARγ gene expression (Fig. 3j). In addition, after 4 weeks of CR, perilipin1 gene expressions and protein level were lower in the R_4_ group compared with the AL_4_ group, which decreased even further after refeeding (*P* < 0.05) (Fig. 3k, l, and m). Collectively, our findings lead to the proposal that CR might be induce and sustain the imbalance of lipogenesis/fat storage capacity during CUGA.

### Changes in gene expression of lipolytic enzymes in VAT during CUGA

**Fig. 4:**
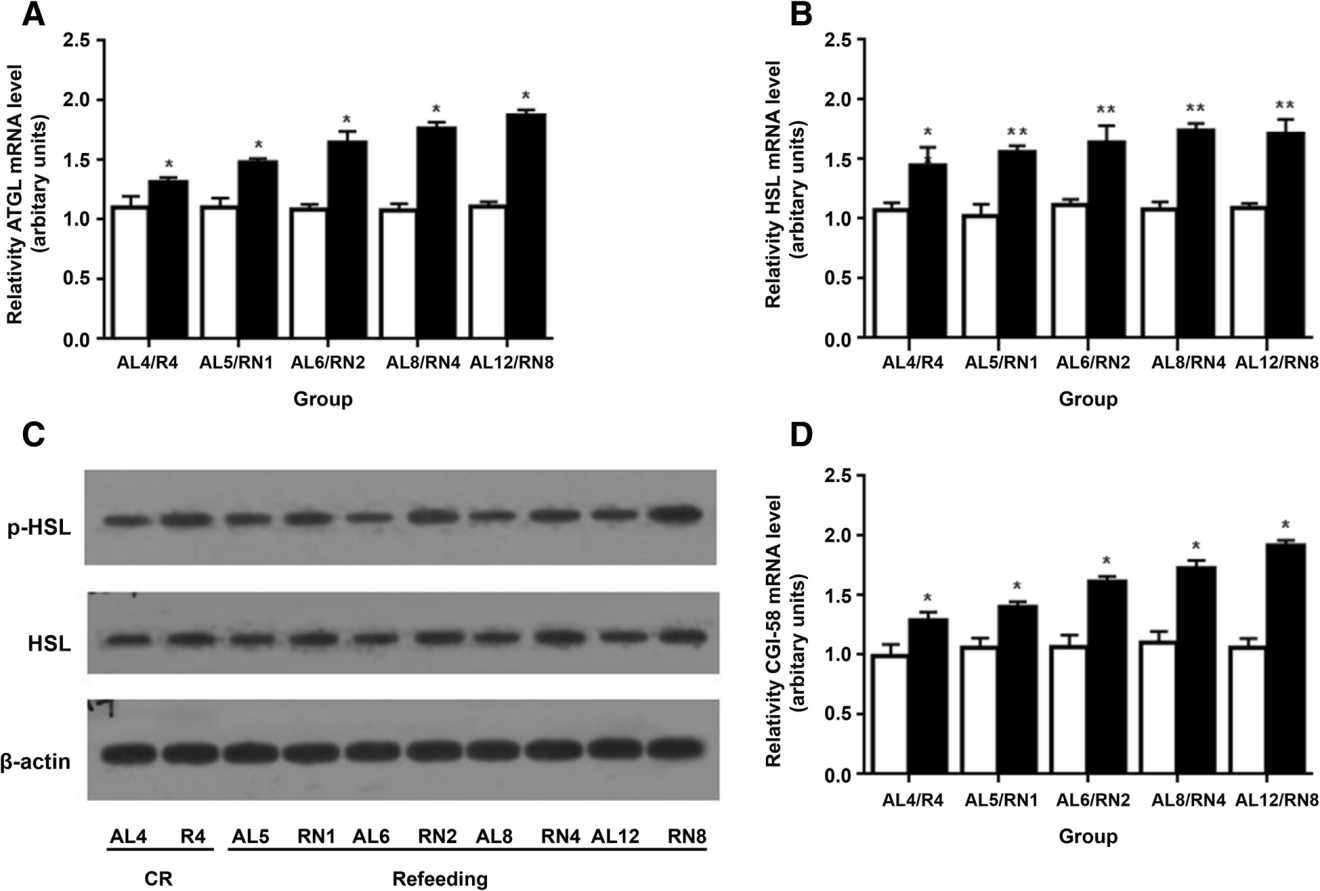
Expression of key genes involved in lipolysis in VAT during CUGA. Quantitative PCR analyzing expression of (**a**) ATGL and (**d**) its co-activator CGI-58. Analysis of HSL (**b**) gene expression. **c** Ratio of HSL/Ser^660^-phosphorylated HSL. Values normalized to β-actin. n = 4/group. White bars, AL groups; black bars, RN groups. *P < 0.05, **P < 0.01 versus the corresponding AL groups

Since ATGL gene and protein expression is highly correlated under several different experimental conditions, we only assayed ATGL mRNA level in this study. As shown in Fig. 4, ATGL gene expression was significantly elevated in the R_4_ group in comparison with the AL_4_ group as a result of 4 weeks of CR (*P* < 0.05). This was more evident in the RN groups than in their controls after refeeding (*P* < 0.01) (Fig. 4a). Comparatively, both gene expression (HSL, *P* < 0.01) and protein levels (Ser^660^-phosphorylated HSL, *P* < 0.01) were also significantly increased in the R_4_ group compared to the AL_4_ group in response to 4 weeks of CR. After refeeding, this was even more apparent in the RN groups than in their controls (Fig. 4b, c). Moreover, expression of CGI-58 was consistent with ATGL gene expression. CGI-58 gene expression was enhanced by CR and increased further after refeeding in the RN groups relative to the controls (*P* < 0.01) (Fig. 4d). In brief, these results indicate that following refeeding, the lipolytic capacity in VAT was enhanced in the RN group, which displayed lipid overflow in their VAT.

### Morphology of perirenal adipose tissue and the distribution of adipocyte size during CUGA

**Fig. 5:**
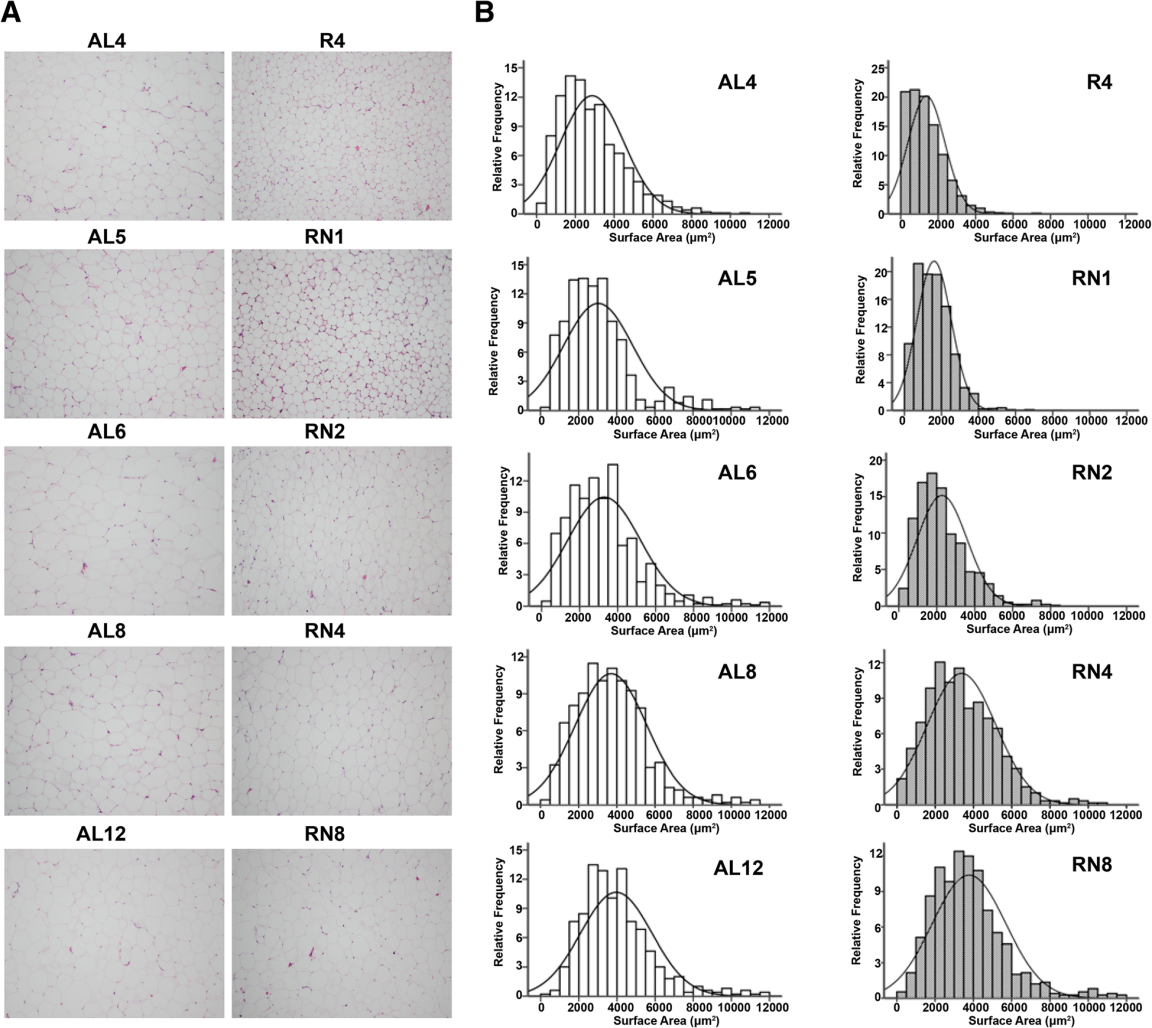
Frequency distribution of surface area of adipocytes in VAT. **a** Morphological detection (× 100) of perirenal adipose tissue to reveal the distribution of adipocyte size by using Hematoxylin/eosin staining. **b** Data obtained by image analysis of histologic sections. Statistical comparison of adipocyte area distribution between RN and AL groups were performed by Kolmogorov-Smirnov tests. n = 4/group. White bars, AL group; grey bars, RN group

The morphology of H&E staining showed that adipocyte size tended to be smaller in the R_4_ group than in the AL_4_ group at the end of CR. After refeeding, the adipocyte size in the RN groups increased gradually over time, but it failed to exceed that of controls even after 8 weeks of refeeding (Fig. 5a). Quantitative measurement of cell profile area revealed that adipocyte size distribution in RN group exhibited a significant shift to the left. Four weeks after CR, the median adipocyte area in the R_4_ group was dramatically smaller than in the AL_4_ group (1369.10 μm^2^ vs. 2867.07 μm^2^, *P* < 0.01). Four weeks after refeeding, adipocyte area distribution in the RN_4_ group still displayed a significant shift to small size relative to controls (3369.00 μm^2^ vs. 3964.60 μm^2^, *P* < 0.05). Even eight weeks after refeeding, the mean adipocyte area in the RN_8_ group could not exceed that of controls (3755.64 μm^2^ vs. 3961.70 μm^2^, *P* = 0.63) (Fig. 5b). Taken together, we observed a persistent shift-to-the-left of adipocyte size distribution accompanied by enhanced lipogenesis during CUGA.

## Discussion

The main findings of this study relate to the mechanisms linking visceral fat accumulation to ectopic lipid deposition and IR in CUGA. The results suggested that the pathophysiological consequences of CUGA are associated with (i) CR-induced and sustained enhancement of lipogenesis in VAT after refeeding, which results in visceral fat accumulation; (ii) CR-induced and sustained mismatch between lipogenesis and fat storage capacity in VAT during refeeding, which leads to ectopic lipid deposition and IR.

In this study, we initially investigated the changes in lipogenic capacity in VAT during CUGA. Our study showed that PPARγ expression in VAT was significantly higher in the R_4_ group than in the AL_4_ group after 4 weeks of CR. A previous study also found that 12-month CR markedly increased PPARγ expression in VAT [9], but another study reported that PPARγ expression in VAT was declined after 2 weeks of CR [28]. These differential changes of PPARγ expression in VAT in response to CR can be attributed to the different duration of CR. The majority of the effects of long-term CR on responsive gene expression can be produced after 4 weeks and the 2-week CR period might be a little short to observe significant effects.

Notably, despite an increase in PPARγ expression in VAT in the R_4_ group, there were no differences in FAS activity and 2-DG uptake between the R_4_ and AL_4_ groups, and AFM% was found to be lower in the R_4_ group, suggesting that nutrition deficiency at the CR stage might be an important reason for the lack of lipogenic capacity in VAT. A similar finding of LPL gene expression further supported this suggestion. It could be hypothesized that CR increases lipogenesis to keep up with the demand to compensate for limited fuel supply, but a previous study suggested that in the context of nutrition shortage, this enhanced lipogenic capacity may be only an enhanced lipogenic potential [42], which will have to be verified in future studies. At the following refeeding period, we observed that PPARγ and LPL expression, FAS activity and 2-DG uptake in VAT in the RN groups were all higher than in controls, concomitant with which AFM% in the RN groups was rapidly increased with the time of CUGA. These results are consistent with previous study for catch-up accumulation of fat rats during 10-day refeeding after CR [28]. Our findings suggested that due to the adequate nutrition after refeeding, the enhanced lipogenic potential in VAT induced by CR transformed into enhanced lipogenic capacity, resulting in a rapid visceral fat accumulation. In a word, these results supported that enhanced lipogenesis might be a crucial factor for initiating and sustaining visceral fat accumulation during CUGA, which was induced by CR, and persisted after refeeding.

Compared with the enhanced lipogenic capacity, the relative decrease in triacylglycerol storage capacity in VAT is an important and surprising finding in this study. Results showed that despite an increase in PPARγ expression in R_4_ VAT after 4 weeks of CR, FSP27 expression was decreased in R_4_ VAT. After refeeding, PPARγ expression persistently increased in RN VAT. On weeks 2 and 8 of refeeding, PPARγ expression in RN VAT was increased to 1.7 and 2.2 times that of controls, respectively. In contrast, FSP27 expression in RN VAT gradually returned to the level of controls until 2 weeks of refeeding. On week 8 of refeeding, FSP27 level in RN VAT increased to 1.29 times that of controls. Thus, although the expression of PPARγ and FSP27 in RN VAT increased with the time of refeeding, FSP27 expression did not follow the increase in PPARγ expression. These results suggested that CR induces the imbalance of PPARγ and FSP27 expression in VAT, which may be an initiating element for the uncoordinated lipogenesis and fat storage capacity in VAT during refeeding. Additionally, we observed a sustained decrease in expression of perilipin1 in VAT in the RN groups, and a persistent shift-to-the-left of adipocyte size distribution accompanied by enhanced lipogenesis during CUGA. These data further supported the imbalance of lipogenesis/fat storage capacity in VAT during CUGA. This imbalance may be especially significant to the pathogenesis of IR in CUGA, as the imbalance of lipogenesis/fat storage capacity prevents synthesized triacylglycerol from efficiently storing in WAT, thus resulting in lipid overflow and ectopic lipid deposition, which causes the development of IR. Our findings lead to the proposal that CR-induced and sustained imbalance of lipogenesis/fat storage capacity might mediate a cross-talk between catch-up accumulation of fat and IR during CUGA.

Interestingly, our results showed that after CR, FSP27 and perilipin1 level in VAT were significantly decreased in the R_4_ group compared with the AL_4_ group, whereas there were increases in the expression of lipolytic-related genes such as ATGL, CGI-58, and HSL in the R_4_ group, suggesting that CR induced lipolysis in VAT and promoted lipid overflow. This is in agreement with numerous previous reports [26, 43, 44]. Nevertheless, we failed to find elevated serum NEFA, ectopic triglyceride deposition and impairment of systemic insulin sensitivity in R_4_ group at this stage. This might be ascribed to the nutrition deficiency, which promotes the release of NEFA from WAT to oxidize for energy in peripheral tissues, thereby preventing lipotoxicity.

In addition to the observation of similar expression profile of ATGL gene and protein of HSL phosphorylation during the following refeeding, we discovered that although the lipogenic capacity in VAT was enhanced in the RN groups, the triglyceride storage capacity was relatively insufficient (slowly increased FSP27 expression and persistently reduced perilipin1 expression), and expression of ATGL and CGI-58 was markedly up-regulated, suggesting that lipolytic capacity in VAT was enhanced in the RN group after refeeding. This is further supported by the result that serum NEFA was significantly higher in the RN groups than in the AL groups at refeeding stage. Herein, during CUGA, RN rats showed increased lipolysis and lipid overflow in VAT. We speculated that despite an increase in triglyceride synthesis in RN VAT during refeeding, triglyceride storage capacity in RN VAT was relatively reduced, thus providing more contact surface area of LD for ATGL and HSL, which combined with up-regulation of ATGL, CGI-58 and HSL phosphorylation and down-regulation of perilipin1 facilitates lipolysis and lipid overflow.

The present study only examined perirenal fat and was considered to be representative of visceral fat, but future studies should directly examine the contribution of all types of visceral adipose tissues (mesenteric, omental, perirenal, and epididymal). In addition, since refeeding depends on an initially inadequate intake, which will produce their own acute and chronic adjustments in metabolism, this suggests that caloric restriction may be the initial reason for imbalance between lipid production and storage capacity. This will have to be further examined in future studies. Finally, given that %body fat and %AFM were increased while the mean cell size decreased, it seems that adipocyte numbers increased in the RN groups, which could be supported by the increase in PPARγ. These small cells might be more insulin-sensitive than those of the AL rats. Furthermore, a greater number of small cells might be expected to provide greater fat storage capacity. This is also a hypothesis that will have to be examined in future studies.

**Fig. 6:**
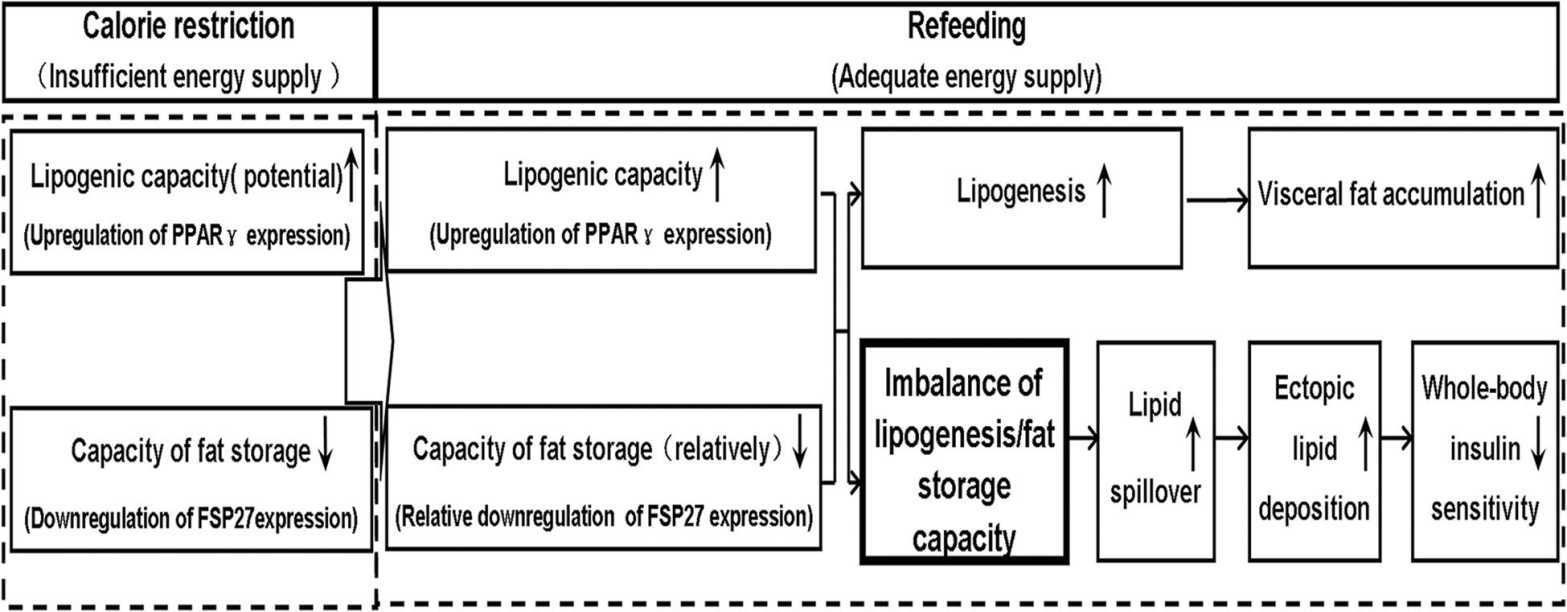
Proposed hypothetical model depicting mechanisms of CUGA-associated IR

Based on our findings, we tentatively propose a hypothetical mechanistic rationale for the development of IR in CUGA. On one hand, at CR stage, in order to effectively compensate the limited energy supply, PPARγ expression increases in VAT, which leads to an increase in lipogenic capacity in VAT (“active storage energy”). At this time, FSP27 expression adaptability down-regulates, and lipid storage capacity of VAT decreases correspondingly. On the other hand, after refeeding, PPARγ expression persistently increases in VAT. At this stage, adequate nutrition supply turns CR-induced lipogenic potential into enhanced lipogenic capacity, causing the rapid accumulation of visceral fat. FSP27 expression cannot correspondingly up-regulate, resulting in relative insufficient capacity of lipid storage. Hence, the change in lipogenesis/lipid storage capacity in VAT adapts to CR and persists even in a new nutritional environment, which leads synthesized triglyceride to exceed triglyceride storage in VAT, causing lipid overflow and the development of IR during CUGA. This study assists us in understanding the underlying mechanisms of CUGA-associated metabolic disorders, and is of significance for the control and prevention of IR-associated disease in developing countries (Fig. 6).

In conclusion, this study reveals part of the mechanisms involved in the development of IR in CUGA from VAT dysfunction as triglyceride depot. The persistent CR-induced imbalance of lipogenesis/fat storage capacity might be responsible for the CUGA-associated metabolic disorders.

## Abbreviations

AFM: Abdominal fat mass
AL: Ad libitum normal chow
ATGL: Adipose triglyceride lipase
BFM: Body fat mass
CGI-58: Comparative gene identification 58
CR: Caloric restriction
CUGA: Catch-up growth in adult
DEXA: Dual-energy X-ray absorptiometry
FAS: Fatty acid synthase
FSP27: Fat-specific protein27
HSL: Hormone-sensitive lipase
IR: Insulin resistance
LBM: Lean body mass
LD: Lipid droplets
LPL: Lipoprotein lipase
NEFA: Non-esterified fatty acid
PPARγ: Peroxisome proliferator-activated receptors γ
SD: Sprague–Dawley
VAT: Visceral adipose tissue
WAT: White adipose tissue
